# Atypical Antipsychotic-Induced Tardive Dyskinesia in a Middle-Aged Schizophrenic Patient: A Case Report

**DOI:** 10.7759/cureus.65663

**Published:** 2024-07-29

**Authors:** Walaa E Mulla

**Affiliations:** 1 Department of Psychiatry, Bahrain Psychiatric Hospital, Manama, BHR

**Keywords:** neuroplasticity, extrapyramidal symptoms, clozapine, valbenazine, movement disorder, schizophrenia, atypical antipsychotics, aripiprazole, tardive dyskinesia

## Abstract

Tardive dyskinesia (TD) is a potentially irreversible movement disorder characterized by involuntary, repetitive movements, most commonly affecting the face, tongue, and extremities. It is primarily associated with the long-term use of first-generation (typical) antipsychotics but can also occur with second-generation (atypical) antipsychotics such as aripiprazole. Despite its lower risk profile, aripiprazole can induce TD, as illustrated by a 45-year-old woman with schizophrenia who developed severe involuntary movements after five years of stable treatment with this medication. Her symptoms, including facial grimacing and choreiform movements, were assessed using the Abnormal Involuntary Movement Scale (AIMS), scoring 18, indicative of moderate to severe TD. Following a switch to clozapine and the addition of valbenazine, a VMAT2 inhibitor, the patient experienced significant symptom reduction and improved quality of life. This case emphasizes the need for ongoing monitoring of TD in patients on long-term antipsychotic therapy, even with atypical agents. Effective management strategies, including medication adjustment and the use of VMAT2 inhibitors, are crucial for optimizing patient outcomes and quality of life. Continued research is needed to better understand and address TD in clinical practice.

## Introduction

Tardive dyskinesia (TD) is a potentially irreversible movement disorder characterized by involuntary, repetitive movements, most commonly affecting the face, tongue, and extremities [1]. It is a well-recognized adverse effect of long-term antipsychotic treatment, particularly with first-generation (typical) antipsychotics. However, TD can also occur with second-generation (atypical) antipsychotics, although the incidence is generally lower [1,2].

The pathophysiology of TD is not fully understood, but it is believed to involve dopamine receptor supersensitivity and neuroplastic changes in the basal ganglia due to prolonged dopamine blockade [1-3]. Risk factors for developing TD include older age, female gender, duration of antipsychotic treatment, and cumulative antipsychotic dose. Additionally, patients with mood disorders or schizophrenia are at higher risk [2,3].

Aripiprazole, a partial dopamine agonist, has been associated with a lower risk of TD compared to other atypical antipsychotics. However, cases of aripiprazole-induced TD have been reported, suggesting that even medications with a lower propensity for causing extrapyramidal symptoms (EPS) can lead to this condition. The management of TD is challenging and primarily involves the reduction or discontinuation of the offending antipsychotic, if feasible. Switching to a different antipsychotic, particularly clozapine, which has a lower risk of TD, can be beneficial. In addition, vesicular monoamine transporter 2 (VMAT2) inhibitors, such as valbenazine and deutetrabenazine, have been approved for the treatment of TD and have shown efficacy in reducing involuntary movements [1-3].

## Case presentation

A 45-year-old woman with a history of schizophrenia presented to the outpatient clinic with a new onset of involuntary movements affecting her face and extremities. Diagnosed with schizophrenia at age 23, the patient had undergone treatment with various antipsychotic medications, including both typical agents (e.g., haloperidol) and atypical agents (e.g., risperidone, quetiapine). For the past five years, she had been maintained on aripiprazole 15 mg daily, which provided stable psychiatric symptom control with minimal adverse effects.

Approximately six months before presentation, the patient began experiencing a gradual onset of involuntary movements. Initially, she noticed facial grimacing and lip-smacking, which later progressed to tongue protrusion and choreiform movements of her hands and feet. These symptoms worsened over time, leading to significant social embarrassment and functional impairment, including difficulties with daily activities and professional responsibilities.

On clinical examination, the patient exhibited prominent oro-bucco-lingual dyskinesia characterized by repetitive facial grimacing and lip-smacking. Additionally, she displayed choreoathetoid movements of the upper and lower extremities, including irregular, twisting motions of her hands and feet. A neurological examination did not reveal any other deficits, and her cognitive function remained intact. Her psychiatric symptoms, including auditory hallucinations and paranoid delusions, were well-managed on her current medication regimen.

The patient's medical history was notable for no substance abuse, head trauma, or other medical conditions that could contribute to her symptoms. Laboratory investigations, including complete blood count, liver function tests, thyroid function tests, and a metabolic panel, were all within normal limits. A brain MRI was performed to exclude structural abnormalities and revealed no significant findings.

Given the clinical presentation and the temporal association with long-term antipsychotic use, a diagnosis of tardive dyskinesia (TD) was made. The Abnormal Involuntary Movement Scale (AIMS) was administered, yielding a score of 18, indicative of moderate to severe TD.

Following a thorough discussion of the potential risks and benefits, the patient agreed to a trial of medication adjustment. Aripiprazole was gradually tapered, and clozapine was introduced as an alternative, given its lower risk of inducing TD. Additionally, the patient was started on valbenazine 40 mg daily, a VMAT2 inhibitor known for its effectiveness in managing TD.

At the three-month follow-up, the patient showed a significant reduction in involuntary movements, with the AIMS score decreasing to 10. She reported substantial improvements in her quality of life and no exacerbation of her psychotic symptoms, demonstrating the efficacy of the combined treatment approach.

## Discussion

TD remains a significant concern in the management of chronic schizophrenia, particularly in patients undergoing long-term antipsychotic treatment. While TD is more commonly associated with first-generation (typical) antipsychotics, it can also occur with second-generation (atypical) agents such as aripiprazole [3,4]. This case highlights that even atypical antipsychotics, which are generally perceived as having a lower risk for TD, are not entirely free from this side effect.

Aripiprazole, a partial dopamine agonist, is often chosen for its relatively favorable side effect profile compared to typical antipsychotics. However, this case underscores that TD can still develop, particularly with prolonged use. The mechanisms underlying TD are believed to involve chronic dopamine receptor blockade and neuroplastic changes in the basal ganglia, leading to supersensitivity of dopamine receptors. Despite aripiprazole’s partial agonist activity, the risk of TD cannot be entirely eliminated [3-5].

This patient’s management involved switching from aripiprazole to clozapine, a medication with a lower risk of inducing TD. Additionally, valbenazine, a VMAT2 inhibitor, was introduced to address tardive dyskinesia. The combination of these strategies resulted in a significant reduction in TD symptoms and improved quality of life for the patient. This approach aligns with current treatment guidelines, which suggest that VMAT2 inhibitors can be effective in managing TD symptoms and that switching to clozapine can be beneficial [2,4].

The findings from this case emphasize the importance of regular monitoring for TD in patients on long-term antipsychotic therapy, regardless of the type of antipsychotic used. Early detection and intervention are critical to managing this condition effectively and improving patient outcomes. Clinicians should consider individual patient risk factors, such as duration of treatment and gender, when evaluating the risk of TD [2-7].

## Conclusions

This case of aripiprazole-induced tardive dyskinesia in a middle-aged woman with schizophrenia underscores the importance of vigilance in monitoring for tardive dyskinesia, even with atypical antipsychotics. The successful management of her condition through the combination of switching to clozapine and initiating valbenazine highlights the efficacy of personalized treatment strategies. It is imperative for clinicians to regularly assess for tardive dyskinesia, consider individual risk factors, and involve patients in their treatment decisions to optimize outcomes and quality of life. Further research into the mechanisms and treatment of tardive dyskinesia remains essential to improve care for affected individuals.
